# Preliminary report on Sichuan golden snub-nosed monkeys (*Rhinopithecus roxellana roxellana*) at Laohegou Nature Reserve, Sichuan, China

**DOI:** 10.1038/s41598-018-34311-z

**Published:** 2018-11-01

**Authors:** Gu Fang, Man Li, Xiao-Jie Liu, Wei-Jia Guo, Yu-Ting Jiang, Zhi-Pang Huang, Shi-Yi Tang, Da-Yong Li, Ji Yu, Tong Jin, Xiao-Geng Liu, Ji-Mei Wang, Sheng Li, Xiao-Guang Qi, Bao-Guo Li

**Affiliations:** 1 College of Life Sciences, Shaanxi Key Laboratory for Animal Conservation, Northwest University, Xi’an, 710069 China; 2 College of Life Sciences, China West Normal University, Nanchong, 637009 China; 3 The Nature Conservancy (TNC) China Program, Beijing, China; 4 The Laohegou Nature Reserve, Mianyang, 622552 China; 5 School of Life Sciences, Peking University, Beijing, 100871 China; 6 Center for Excellence in Animal Evolution and Genetics, Chinese Academy of Sciences, Kunming, 650223 China

## Abstract

Comparative studies of subspecies under different ecological environments offer insights into intraspecies evolutionary adaptive mechanisms. Golden snub-nosed monkeys (*Rhinopithecus roxellana*) include three subspecies in China classified mainly by their morphological variations: *R. r. roxellana* (Sichuan and Gansu province)*, R. r. qinlingensis* (Shaanxi province) and *R. r. hubeiensis* (Hubei province). These three subspecies live in three isolated area with different environments. Past works focused on the last two subspecies, but little information of habitat and behaviors of the nominated subspecies (*R. r. roxellana*) is available to date. We conducted a two-year study on the diet, activity budget, home range and social organization of 4 herds of *R. r. roxellana*, based on a total of 106 days’ observation in Laohegou (LHG) Nature Reserve, Sichuan province. By using scan sampling method, our results suggest that the *R. r roxellana* feeds predominantly on leaves (77.5%), and spends more time feeding (40.0%) and resting (27.0%) while compared to the other two subspecies. Kernel Density Estimation Method based on GPS technology confirms that *R. r roxellana* has relatively larger home ranges (49.1 km^2^). The unit size (8.3 ± 3.5 individuals) of *R. r roxellana* is also smaller. Therefore, it is possible that differences in food availability in relation to habitats have important impacts on the feeding strategy and social system of the golden snub-nosed monkey. These results provide data to further explore intraspecific adaptations of living primates.

## Introduction

Species in different parts of their geographic range may evolve different adaptations to environmental variations, and are likely to vary in behavior^1^. Due to natural processes and human interference, some wide-ranging species have been confined into isolated habitats with different ecological conditions^2^.

Snub-nosed monkeys (*Rhinopithecus* spp.) are an endangered Asian colobine that lives in temperate forests of mountainous highlands. This genus contains 5 species. Of these, the golden snub-nosed monkey (*Rhinopithecus roxellana*), with the northernmost habitat of all colobines^3^, was once widely distributed in China^2^. Unfortunately, most of their populations have vanished under the influence of increased human activities during past thousand years^2,4,5^. Deteriorating environments and accelerated deforestation restricted this species to fragmented and limited areas under the effect of geographic isolation^2,6–9^, which led to differentiation into three subspecies: *Rhinopithecus roxellana roxellana* (in Minshan mountains of Sichuan and Gansu province)*, R. r. qinlingensis* (in Qinling mountains of Shaanxi province) *and R. r. hubeiensis* (in Shennongjia mountains of Hubei province)^10,11^.

These isolated habitats exhibit different environmental conditions. For instance, the habitat of *R. r. qinlingensis* is a mostly deciduous broadleaf forest area in Qinling, Shaanxi^8^, while *R. r. hubeiensis* in Shennongjia, Hubei lives in a mixed coniferous broadleaf forest area^12^. The *R. r. roxellana* population in Sichuan is distributed in areas with deciduous and evergreen broadleaf forest^11^. The differentiation of these three subspecies provides an example and offers insights to explore the adaptive mechanism of intraspecies evolutionary radiation.

Previous studies mainly focused on the first two subspecies, *R. r. qinlingensis* and *R. r. hubeiensis*. Studies revealed behavioral differentiation between those two subspecies, such as foraging preference divergence^4,13–15^. However, due to the precipitous mountain habitat, little knowledge is available on the nominate subspecies (*R. r. roxellana*) in Sichuan, even though the first specimen of this species was discovered there and named in 1870 A.D.^11^. These monkeys are shy of humans and move rapidly across steep cliffs and deep gorges, which make them difficult to follow and study. Owe to this lack of the basic information, how golden snub-nosed monkeys shaped to varied environments still remains unclear.

We conducted a two-year study based on field observation of *R. r. roxellana* in Laohegou National Nature Reserve in Sichuan province and report here the information for this subspecies. Based on the behavior observation, we conducted a study on the diet, activity budget and social organization of *R. r. roxellana*. With the assistance of remote sensing and global positioning system (GPS) technology, we confirmed the home range of golden snub-nosed monkeys in Laohegou, Sichuan. Here, we present the first detailed data on *R. r. roxellana* from the wild. Our results would provide evidence to further explore intraspecific adaptations in varied habitats in primates.

## Materials and Methods

### Study area

We carried out this study in Laohegou (LHG) Nature Reserve in Minshan Mountains (104°32′ −104°45′ E, 32°25′ −32°36′ N), Pingwu county, Mianyang city, Sichuan province, China. Based on data of China Meteorological Administration (Website: http://www.cma.gov.cn, in Chinese), climate in this area (1,100–3,100 m asl.) has a subtropical monsoon climate with a cold and dry winter and cool and moist summer. Annual average temperature is 14.7 °C (ranged from −7–37 °C). The relative humidity ranges between 60–80%, and annual average sunshine duration ranges between 867.2–1289.4 hours. The study area has a high frequency of rainy days especially in spring, but the amount of daily rainfall is small (annual precipitation: 760.4 mm–1230.5 mm). In this study, we defined seasons as follows^16^: spring (Mar. 6–Jun. 1), summer (Jun. 2–Aug. 27), autumn (Aug. 28–Nov. 22), and winter (Nov. 23–Mar. 5).

### Study animals

The basic composition of golden snub-nosed monkey society is one male multi-female plus offspring (OMU)^3^. OMUs do not avoid each other, but move, forage and rest together to form a breeding band^17^. Young male normally emigrate from their natal groups before sexual maturity, and form all male units, several of which coalesce into an all male band (AMB)^18,19^. All male band follows around the breeding band, all together comprising a herd^18^. These different social levels represent socially and spatially distinct components of the multilevel society^18,20^.

**Figure 1:**
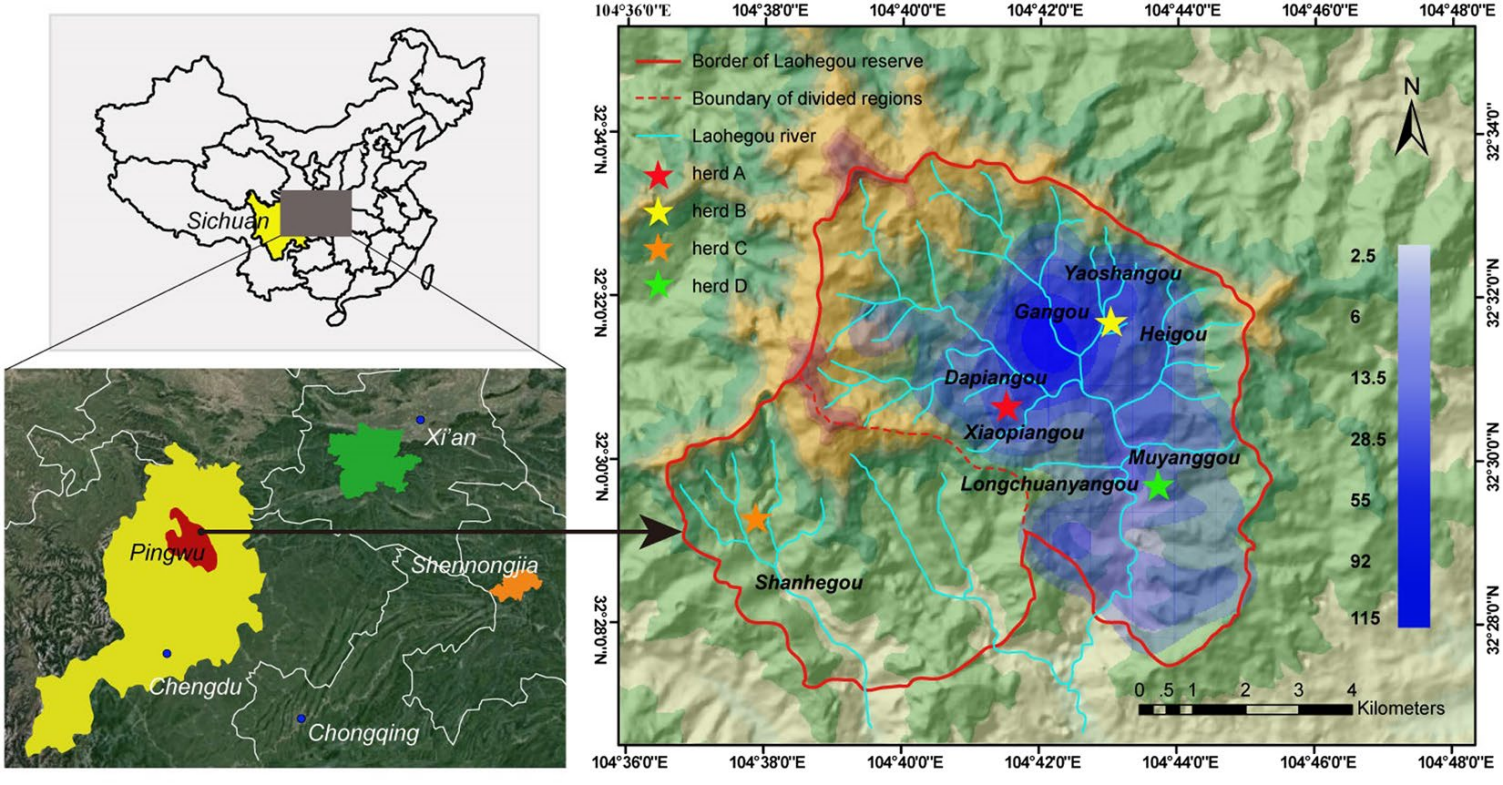
Home range of *R. r. roxellana* in Laohegou, Sichuan province. The home range size was calculated by Kernel Density Estimation Method, with search radius of 1100 meters and total of 869 GPS points. The area filled with blue represents the area of the home range area (49.1 km^2^) used by the herd B (distributed in Gan-gou, Yao-shan-gou and Hei-gou) in Laohegou troop. The figure was processed by ArcGIS®V10.2 (ESRI, Redlands, California). The satellite imagery is provided by a Chinese publicly-accessible website, Geospatial Data Cloud site (Computer Network Information Center, Chinese Academy of Sciences, http://www.gscloud.cn, copyright permission for public posting).

According to previous survey and field research, the staff of the Laohegou Nature Reserve identified 4 herds of monkeys during their daily patrols, each herd has a relatively independent home range (government report: Improvement of golden snub-nosed monkey at Laohegou, in Chinese), including herd A (distributed in Da-pian-gou and Xiao-pian-gou valley), herd B (distributed in Gan-gou, Yao-shan-gou and Hei-gou valley), herd C (distributed in Shan-he-gou valley) and herd D (distributed in Mu-yang-gou and Long-chuan-yan-gou valley) (Fig. 1).

During our observation, herd B was the easiest to directly observe, due to less precipitous terrain. Although we could not identify individual monkey, we recorded basic information on recognition of the band and unit level, which allowed us to estimate the home range of herd B. Herd B included about 130 individuals, including 99 individuals in the breeding band and 31 individuals in the all-male band. We collected home range data from herds A, B, and D.

### Data Collection and Analysis

We conducted two years of observations from Mar. 2013 to Jun., 2015. Due to the precipitous terrain, we collected available data from Mar. 26, 2013 to May 16, 2013 and Apr. 9, 2015 to Jun. 1, 2015 (106 days), so spring was our main field season. We categorized individuals into six age/sex classes: adult males (more than 7 years old), adult females (more than 5 years old), sub-adult males (5–7 years old), sub-adult females (3–4 years old), juveniles (1–3 years old), infants (less than 1 year old)^3,21^.

Home range data were collected by both direct observation and indirect record method. For the direct observations, we conducted the daily patrol, and when we observed the monkeys, we followed and recorded the GPS data of the location. If the direct observation is not possible, and also in order to expand our data, we used infrared cameras and traces judgment (fresh feces or food residues) to perform the indirect observation. If we observed the fresh feces or residues, we recorded its GPS point. The infrared cameras were not relocated during the study. If the cameras recorded the monkeys, we documented its coordinate as the GPS point. Totally 869 GPS points were recorded, including 52 points (5.8%) from 21 infrared cameras. We input the GPS points to the map of LHG via ArcGIS® V10.2 (ESRI, Redlands, California). Kernel Density Estimation Method^22^ was used to divide the irregular and concentric graphic into six layers. The home range sizes were then calculated from the size of the outermost layer area. The pairwise comparisons of tests were performed on a web version of MEDCALC.

For the activity budge data collection, we record daytime activity data of the monkeys in herd B during the direct observation. We used single-tube telescope (LEICA® APO-TELEVID 82) to observe monkeys with distances ranging from 300 to 600 m away from the target individuals. Targets were selected via *ad libitum* sampling and the activity patterns were recorded via scan sampling methods with a ten-minute interval. We categorized activity patterns into four classes: moving (walking on the tree or swinging from tree to tree), foraging (searching and eating food items), resting (sitting or leaning on the tree) and other behaviors (such as grooming or playing)^23,24^. We collected total 4733 records. Data from infants were excluded since their behaviors were not independent. We calculated percentage of feeding time was calculated as number of scans where feeding was recorded/total number of scans.

During our scan, when an animal was foraging, we recorded the food items consumed and classified as leaves, buds, flowers, fruits, barks, lichens and moss. Among total 1893 foraging points, we also identified the name of the plant species ingested for 1244 points. The percentage of time monkey spent feeding on item *X* was calculated from: number of points where item *X* was recorded/total number of foraging points.

### Ethics statements

Research protocols for the study was granted by the Chinese Academy of Sciences, complied with the principles approved by animal care committees of the Wildlife Protection Society of Sichuan Province, China, and adhered to the regulatory requirements of Laohegou National Reserve, China, and to the American Society of Primatologists principles for the ethical treatment of primates.

## Results

### Diet

**Figure 2:**
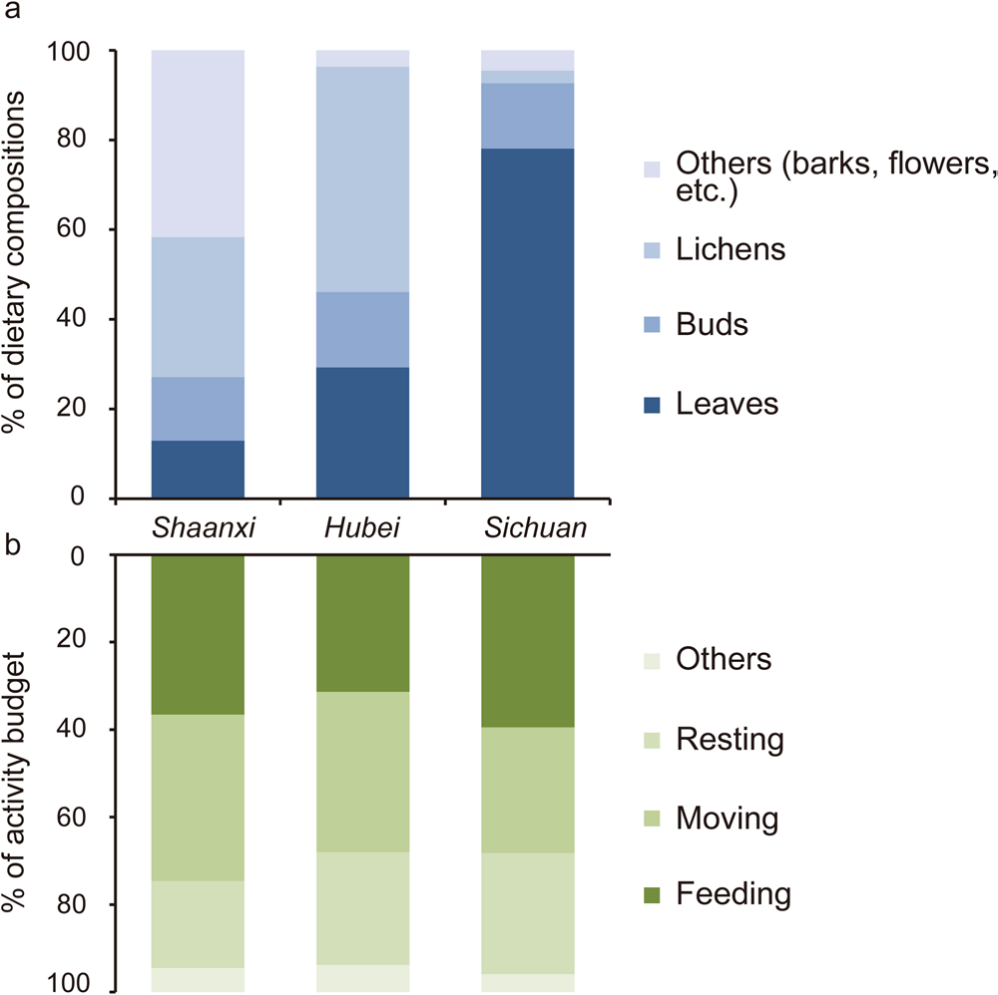
(**a**) Dietary compositions of *R. roxellana* in Shaanxi, Hubei and Sichuan. (**b**) Activity budget of *R. roxellana* in Shaanxi, Hubei and Sichuan. Different color indicated different proportion of food intake/activity budget of *R. roxellana*.

Golden snub-nosed monkeys in LHG spent 77.5% of their foraging time on leaves, 14.5% on buds and 3.2% on fruits. The time they spent on lichens was only 2.8%. Flowers, barks and moss accounted for 0.8%, 0.2% and 0.2% of their diet respectively (Table 1, Fig. 2a). During the observation we found they consumed a total of 12 different plant species, and the taking of *Sorbus zahlbruckneri Schneid* (60.3%) and *IIex polyneura* (18.3%) occupied a large percentage of their feeding time (Table 2). Pairwise comparisons indicated the golden snub-nosed monkey spent significantly more time consuming leaves and less time consuming lichens in Sichuan than the other populations studied in Shaanxi and Hubei (Time consuming leaves, Sichuan-Shaanxi: *Z* = 83.850, *P* < 0.0001; Sichuan-Hubei: *Z* = 46.076, *P* < 0.0001; Time consuming lichens, Sichuan-Shaanxi: *Z* = 26.670, *P* < 0.0001; Sichuan-Hubei: *Z* = 41.247, *P* < 0.0001). This unique dietary pattern was found different from any other populations. In Shaanxi, they feed primarily on barks (38.1%) and lichens (31.2%). In Hubei, lichens (50.2%) were their main food item.

**Table 1:** Dietary compositions of *R. roxellana* in Shaanxi, Hubei and Sichuan (spring).

Dietary components (%)	Leaves	Buds	Fruits	Lichens	Flowers	Barks	Moss	Others
Shaanxi^a^	12.9	14.2	0	31.2	0	38.1	0	3.6
Hubei^b^	29.3	16.8	0	50.2	3.2	0	0	0.5
Sichuan	77.5	14.5	3.2	2.8	0.8	0.2	0.2	0.8

^a^Guo *et al*., 2007, ^b^Liu *et al*., 2016.

**Table 2:** Percentage of time spent feeding on different food species in Sichuan (spring).

Species	Family	Parts consumed	Percentage (%)
*Sorbus zahlbruckneri Schneid*	Rosaceae	buds, leaves	60.3
*IIex polyneura*	Aquifoliaceae	buds, leaves, flowers	18.3
*Toxicodendron verniciflum*	Anacardiaceae	buds, leaves	7.0
*Vitis quinquangularis Rehd*.	Vitaceae	leaves	6.9
*Betula alnoides*	Betulaceae	buds, leaves, barks	2.5
*Cerasus szechuanica*	Rosaceae	leaves, fruits	2.0
*Acer elegantulum*	Loranthaceae	leaves	0.6
*Populus*	Salicaceae	leaves	0.4
*Sorbus megalocarpa*	Rosaceae	flowers	0.3
*Loranthus tanakae*	Loranthaceae	leaves	0.2
*Loranthus Jacq*.	Loranthaceae	leaves	0.1
Unable to identify	/	leaves	1.4

### Activity budget

The golden snub-nosed monkey of herd B spent 40.0% of the daytime feeding, 26.8% moving, 27.0% resting, and 6.2% for other activities, including grooming and playing (Table 3; Fig. 2b). Monkeys from the Sichuan population spent a significantly greater proportion of their time feeding than monkeys from Shaanxi and Hubei (Sichuan-Shaanxi: *Z* = 4.856, *P* < 0.0001; Sichuan-Hubei: *Z* = 42.124, *P* < 0.0001). The moving time was significantly less in Sichuan than in Shaanxi and Hubei (Sichuan-Shaanxi: *Z* = 15.874, *P* < 0.0001; Sichuan-Hubei: *Z* = 22.24, *P* < 0.0001).

**Table 3:** Activity budget of *R. roxellana* in Shaanxi, Hubei and Sichuan (spring).

Activity (%)	Feeding	Moving	Resting	Others	Group size
Shaanxi^a^	36.6	38.0	19.9	5.5	112
Hubei^b^	17.0	42.8	8.7	31.5	120–140
Sichuan	40.0	26.8	27.0	6.2	130

^a^Guo *et al*., 2007, ^b^Li, 2009.

**Figure 3:**
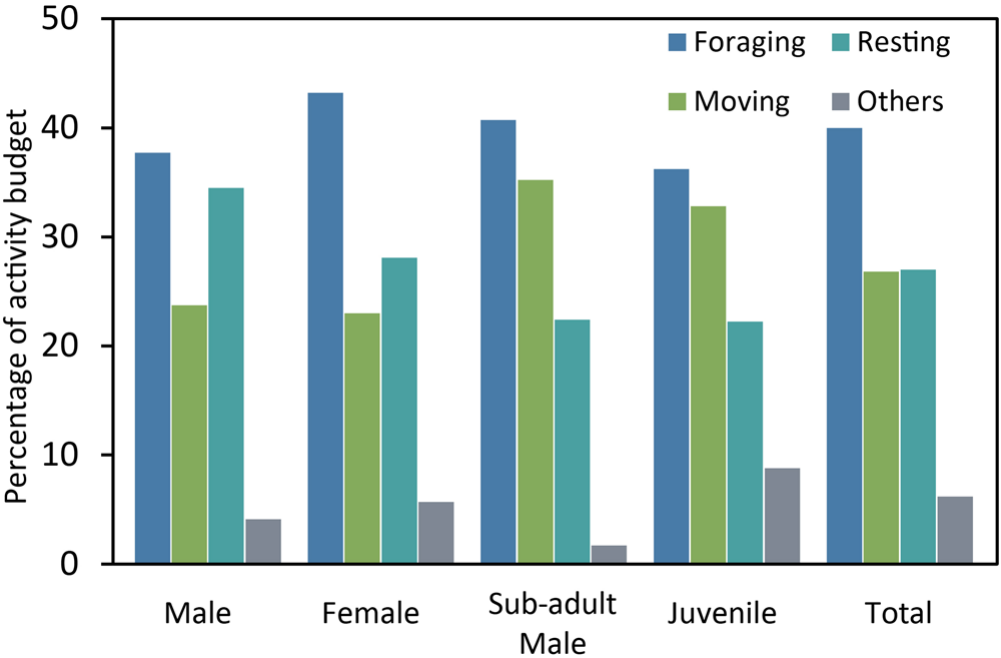
Variation in the activity budget among age-sex classes of *R. r. roxellana* in Laohegou, Sichuan province.

The activity budget among age-sex classes is shown in Fig. 3. Among all the age-sex classes, the golden snub-nosed monkey spent more time foraging than any other activities (Fig. 3). During the daytime, the females spent the highest proportion of their times on foraging compared with other age-sex classes (43.0%), the males occupied the highest proportion for resting (34.0%), the sub-adult males occupied the highest proportion for moving (35.0%). And the juveniles spent the highest proportion of their times on playing or grooming (9.0%). In spring, feeding behavior got into three peaks at 10:00–11:00 h, 14:00–15:00 h and 16:00–17:00 h. Resting was largely concentrated in on two periods, 11:00–13:00 h and 15:00–16:00 h. Moving mainly appeared in 09:00–10:00 h, by leaving from the sleeping site to the feeding site.

### Home range

In LHG, the golden snub-nosed monkey mainly lived in the broadleaf forest. The average elevation of their distribution area was 1853m (90.0% of the habitat was between 1400–2200 m, with 6.8% between 2200–3100 m and 3.2% between 1100–1400 m). Based on the results of Kernel Density Estimation Method^22^, the home range size of herd A, B, and D was 49.1 km^2^ (Fig. 1). Home range of herd B was 12.3 km^2^, and population density was 10.6 individuals/km^2^, with total of 130 individuals.

### Social organization

The herd B contained breeding band (BB) and all-male band (AMB). The BB was composed of 12 one male units (OMUs), with a 1:4.2 mature sex ratio (M/F) (Table 4). The size of the OMUs ranged from 3 to 14 individuals (mean = 8.3 ± 3.5), with 2.7 ± 1.3 adult females, 1.5 ± 1.2 sub-adult females, 2.6 ± 1.4 juveniles and 0.5 ± 0.5 infants (Table 5). There were 31 males in the AMB, including 6 adult males, 10 sub-adult males, and 15 juveniles. At the same time, we found that a solitary monkey existed in some area.

**Table 4:** Social composition of *R. r. roxellana* in herd B, Laohegou Nature Reserve, Sichuan (spring).

Group number	BB						AMB			
	M	F	SF	J	I	Total	M	SM	J	Total
1	1	2	2	4	0	9	6	10	15	31
2	1	1	0	1	0	3				
3	1	4	4	5	0	14				
4	1	2	1	2	0	6				
5	1	5	2	4	1	13				
6	1	1	1	1	0	4				
7	1	4	2	2	1	10				
8	1	4	0	4	1	10				
9	1	3	3	3	1	11				
10	1	2	1	2	1	7				
11	1	2	1	2	0	6				
12	1	2	1	1	1	6				
Total	99						31			
Total	130									

Herd B means monkey distributed in Gan-gou, Yao-shan-gou and Hei-gou.

BB = breeding band, AMB = all-male band.

M = adult male, SM = sub-adult male, F = adult female, SF = sub-adult female, J = juvenile, I = infant.

**Table 5:** Comparison of unit composition in Shaanxi, Hubei and Sichuan (spring).

Sites	Unit size	Adult female	Sub-adult female	Juvenile	Infant
Zhouzhi, Shaanxi^a^	11.1 ± 2.0 (5–14)	3.3 ± 0.9* (2–5)	1.1 ± 0.6* (0–2)	2.0 ± 0.9* (1–4)	1.0 ± 0.8* (0–2)
Shennongjia, Hubei^b^	11.8 ± 4.6* (5–17)	4.4 ± 2.1* (2–8)	0.5 ± 0.3* (0–1)	2.5 ± 1.4* (1–5)	3.7 ± 3.3* (0–10)
Laohegou, Sichuan	8.3 ± 3.5 (3–14)	2.7 ± 1.3 (1–5)	1.5 ± 1.2 (0–4)	2.6 ± 1.4 (1–5)	0.5 ± 0.5 (0–1)

^a^Zhang *et al*. (2006), ^b^Luo Fang (2010), *The whole year’s data, e.g.: 11.1 ± 2.0 (5–14), mean = 11.1, SD = 2.0, minimum = 5, maximum = 14.

## Discussion

Our result suggested leaves were the largest dietary component for *R. r. roxellana* in spring. Golden snub-nosed monkeys in Sichuan would spend more time on feeding and resting, instead of moving. The home range is 49.1 km^2^, while the unit size of *R. r. roxellana* is relatively smaller (8.3 ± 3.5 individuals, ranged from 3 to 14). Our result might provide basic and valuable information to compare between subspecies under different ecological factors.

Dietary composition makes a regional difference within species in our study. In spring, leaves (*Sorbus zahlbruckneri Schneid*, *IIex polyneura, et al*.) were the largest dietary component for the populations in Sichuan, while monkeys primarily fed on barks and lichens in Shaanxi, and lichens for the population in Hubei.

Intraspecific variation of diet might be linked to the availability of potential food resources^25^. Different patterns of food availability might induce various food preferences^26,27^. For *R. roxellana*, the population in Sichuan distribute in areas with deciduous and evergreen broadleaf forest where trees sprouted new leaves earlier in spring compared with habitats for other two populations. At the field site of Zhouzhi, Shaanxi, the habitat for *R. r. qinlingensis* is a mostly deciduous broadleaf forest area^13^. At the field site for *R. r. hubeiensis* at Shennongjia, Hubei, monkeys lives in a mixed coniferous broadleaf forest area^28^. The budding time and forest type for *R. r. roxellana* may provide high availability of taking foliage as important food resource.

Foliage contains relatively higher concentrations of protein compared with other diet composition of *R. roxellana*^27^. As a key nutrient, protein provides enough energy to keep healthy for living organisms. Golden snub-nosed monkey prefer to feed on leaves of trees rather than other food sources. Lichens or barks, as alternative food sources, would only been taken as a proximate response to ecological conditions with deficiency of highly nutritious food^29^.

For achievement of a balance between food intake and consumption, primates acquire nutrition and energy from foraging various food to ensure enough fuel for the digestive process and maintenance of a functioning body^30^. Therefore, monkeys perform different activity budget and energy investment based on various environmental and social conditions^28,31^. A number of studies show that activity budget is largely influenced by diet^26,29,32–36^. For instance, folivorous monkeys, such as langurs, are found to spend considerable time feeding on bulky leaves, and resting to allow for the digestion and fermentation process of the cellulose in their plant diet^37–40^.

In our study, compared with other two subspecies, the feeding time and resting time of *R. r. roxellana* are both increased, while moving time is decreased, which is consistent with the folivorous diet in this population. *R. r. roxellana* might have to spend more time in resting because they consumed more indigestible fiber from foliage to be digested^27^. In addition, times spent on locomotion and food seeking activities can be reduced under habitat with high levels of food abundance. Thus, our result might suggest that the activity budget of the golden snub-nosed monkey would vary between different ecological conditions, since the diet differentiation.

Compared to 22.5 km^2^ of home range in Zhouzhi, Shaanxi^13^, and 40 km^2^ in Shennongjia, Hubei^41^, *R. r. roxellana* in Laohegou, Sichuan had the largest home range (49.1 km^2^). One possible reason to explain this inconsistency may be ascribed to the special attributes of our study site. The leaves contained in the diet of *R. r. roxellana* were scattered and dispersed, which means individuals and groups have to expand their foraging areas to cater for the requirement of energy and nutrition. However, under this assumption, *R. r. roxellana* may spend more time on moving, which is contrast to our result about activity budget. More data and evidences are needed to explore the specific mechanism in future.

Population density in Sichuan is the highest with 10.6 individuals per km^2^, comparing with other populations: 4.0 individuals per km^2^ in Shaanxi^13^ and 4.8 individuals per km^2^ in Hubei^41^. As population density, basically determined by the capability of a habitat to provide sufficient energy and nutrition for the corresponding population, were also found as another dependent factor affected by geographic features and habitat ecology^42^. Compared with other two populations, we suggest that the food abundant of the habitat in Sichuan may be greater. Thus, it can be capable of supporting a larger number of individuals within a particular range.

We used whole year data in species in Hubei and Shaanxi, since previous studies suggested social combination of golden snub-nosed monkeys remained stable for several months, even years^21^. Our result suggested that unit size and social composition were different among these three subspecies of golden snub-nosed monkeys (Table 5). In our field site of Sichuan, we found larger numbers of social units. The unit size of *R. r. roxellana* is relatively smaller (8.3 ± 3.5, ranged from 3 to 14), compared to other subspecies in Hubei and Shaanxi.

The social organization of primates would be influenced by multiple ecological factors. One possible reason to explain this smaller unit size is the food abundance in habitat of Sichuan, which would lead to males occupy relatively average resource. Since in *R. roxellana*, female choice would initial and impact the male takeover^43^, females would have more options to choose with such equally distributed resource. Under this circumstance, numbers of one male unit would increase and the group size would be smaller. However, it is hard to determine what specific factor and mechanism influenced the smaller unit size in our case. More evidence is needed to test relevant hypothesis. Our result suggested that the pattern of the social organization would show differentiation under different ecological condition, even in the same species.

In conclusion, our study firstly provides detailed data on diet, activity budget, home range and social organization of *R. r. roxellana* under different habitats, compared with other two subspecies. Our result might provide evidence that the behavioral and dietary flexibility of *R. roxellana* enables the golden snub-nosed monkey to survive in different conditions. These results may contribute to an improved understanding of intraspecific adaptations to varied environments and flexible behavioral strategies of primates.

## Data Availability

The datasets generated during and/or analysed during the current study are available from the corresponding author on reasonable request.
